# Design of a virtual data shelf to effectively explore a large database of 3D medical surface models in VR

**DOI:** 10.1007/s11548-023-02851-z

**Published:** 2023-03-03

**Authors:** M. Allgaier, L. Spitz, D. Behme, A. Mpotsaris, P. Berg, B. Preim, S. Saalfeld

**Affiliations:** 1 Department of Simulation and Graphics, Otto-von-Guericke University, Magdeburg, Germany; 2 Forschungscampus STIMULATE, Magdeburg, Germany; 3 Department of Neuroradiology, Otto-von-Guericke University, Magdeburg, Germany; 4 Institute for Diagnostic and Interventional Radiology and Neuroradiology, Munich Clinic Harlaching, Munich, Germany; 5 Institute of Fluid Dynamics and Thermodynamics, Otto-von-Guericke University, Magdeburg, Germany

**Keywords:** Virtual reality, Visualization techniques, Database visualization

## Abstract

**Purpose:**

Medical researchers deal with a large amount of patient data to improve future treatment decisions and come up with new hypotheses. To facilitate working with a large database containing many patients and parameters, we propose a *virtual data shelf*, displaying the 3D anatomical surface models in an immersive VR environment.

**Methods:**

Thereby, different functionalities such as sorting, filtering and finding similar cases are included. To provide an appropriate layout and arrangement of 3D models that optimally supports working with the database, three layouts (flat, curved and spherical) and two distances are evaluated. A broad audience study with 61 participants was conducted to compare the different layouts based on their ease of interaction, to get an overview and to explore single cases. Medical experts additionally evaluated medical use cases.

**Results:**

The study revealed that the flat layout with small distance is significantly faster in providing an overview. Applying the virtual data shelf to the medical use case intracranial aneurysms, qualitative expert feedback with two neuroradiologists and two neurosurgeons was gathered. Most of the surgeons preferred the curved and spherical layouts.

**Conclusion:**

Our tool combines benefits of two data management metaphors, resulting in an efficient way to work with a large database of 3D models in VR. The evaluation gives insight into benefits of layouts as well as possible use cases in medical research.

## Introduction

Medical research often involves the investigation and evaluation of large medical databases. To facilitate a link between the numerical data, e.g., blood pressure, and the actual anatomy as well as a visual analysis, 3D surface models of the anatomy should be integrated. Thereby, a meaningful arrangement of the models is desirable. Virtual reality (VR) offers more and different possibilities to visualize and explore data than conventional monitors. This enables a better examination of object shapes using intuitive interactions and improved depth perception [1]. The benefits of VR when dealing with anatomical 3D models were demonstrated in different medical applications [2, 3]. To exploit these benefits, we introduce a *virtual data shelf* to intuitively arrange 3D objects, including functionalities to support the user in generating new hypotheses. We were inspired by Schott et al. [4], who presented virtual organ shelves motivated by shelves in surgical departments.

Medical researchers often have to deal with an abundance of patient data comprising various medical parameters that influence whether and how treatment should be carried out. After getting an overview of the relevant data, various characteristics of the patient data can be explored and researchers can come up with new hypotheses. Working with the database, e.g., finding similar cases, gets more and more difficult the more parameters and patient data are included. These tasks are enabled by our shelf through a head-mounted display (HMD), under which corrective eyewear could be worn, and thus, users maintained their day-to-day vision quality.

However, to facilitate these tasks, the anatomical objects have to be visually arranged in a proper way. To provide the most suitable visual arrangement, we introduced three layouts: *flat*, *curved* and *spherical*. These are compared within studies in an immersive VR environment based on two main tasks: overview and exploration. A pilot study determined suitable shelf characteristics. A quantitative study investigates the suitability of the layouts regarding the two tasks using an attentive and a pre-attentive task to simulate them. In a qualitative study, we aim at identifying medical use cases and their requirements.

## Related work

Ens et al. [5] present a *personal cockpit* including horizontal and vertical curvature, where the user can switch between several everyday applications. A $40^{\circ }$ field of view was deemed sufficient as the tasks rely on the eyes’ foveal region, which is even smaller. The *cockpit* layout was a $4\,\times \,4$ world-fixed matrix of displays, with which the participants were significantly faster than with view-fixed techniques. Furthermore, the *cockpit* was easy and fast to navigate.

Gao et al. [6] present an amphitheater with varying *egocentric distance-based item sizing* (EDIS), where item size is adjusted according to the distance. Besides comparing different levels of EDIS based on a retrieval and recall task, they found that additional location-fixed and user-defined visual landmarks are useful for a set of 54 items. They used a $110^{\circ }$ field of view.

Cao et al. [7] propose to display a 2D dataset on a curved display where the single items are both arranged in a circular layout and curved by themselves. This work solely used 2D information.

In contrast, Liu et al. [8] compared arrangements of small multiples using the *shelf metaphor* [9] to enhance spatial memory by providing a clear horizontal and vertical alignment. Small multiples are arranged as a tiled display of multiple of the same visualization, and a metaphor describes the use of main attributes of real world objects, like a shelf, in another context to ease usability through integration of familiar interactions. In two studies, they compared flat, quarter, half and full circle layouts with horizontal curvature. The flat shelf was faster and more accurate with a small number of multiples, and while the half circle layout was preferred, there were no differences with many multiples. Our layouts include no curvature, horizontal curvature, and vertical curvature, which was mentioned but not implemented by Liu et al. [8]. The shelf metaphor was also used to display livers in a VR anatomy training application [4]. Here, several virtual shelves can be stacked to build up a *library*.

Another work in immersive analytics, where visual analytics and virtual reality is combined [10], is presented by Satriadi et al. [11]. They used a qualitative approach to investigate how users arrange hierarchical views. As a result, a spherical cap layout is preferred by most users.

A recent study from Takashina et al. [12] evaluated curved virtual interactive surfaces and their operational efficiency with varying curvature, size (up to $27\,\times \,15$) and presentation distance. Based on those parameters, they analyzed target selection speed and error rate. Curved surfaces performed best regarding speed, and a larger curvature radius meant a larger error. A flat surface performed worst due to perspective distortion at the edges, but when a search task was added, it performed fastest, possibly due to seeing all objects at once, whereas curved surfaces have out-of-sight targets.

**Table 1:** Comparison of related work approaches

Authors	Concept	Content	Number of items	Curvature
Ens et al. [5]	Personal cockpit	2D interfaces in 3D environment	4*x*4	Horizontal $+$ vertical
Gao et al. [6]	EDIS	2D icons in 3D environment	6*x*9	Horizontal
Cao et al. [7]	Curved display	2D interface elements in 3D environment	N/A	Horizontal
Liu et al. [8]	Small multiples shelf metaphor	3D statistical visualizations	12 (3*x*4, 4*x*3, 2*x*6)	None, horizontal
Takashina et al. [12]	Curved virtual interfaces	2D grid on surface in 3D environment	27*x*15	None, horizontal
Presented	Virtual data shelf	3D anatomical models	6*x*11	None, horizontal, horizontal $+$ vertical

In contrast to the above-mentioned approaches, our *virtual data shelf* provides three layouts to display complex 3D objects in an immersive environment. The comparison to related work is presented in Table 1. The shelf is scalable by scrolling, providing an uncluttered way to work with a larger database since a manageable subset is displayed. The user is also supported by, for example, filtering and sorting as well as automatically finding similar objects. Furthermore, the *virtual data shelf* is specialized for medical research in the area of intracranial aneurysms, but might also be adapted to other medical applications or non-medical applications. This adaption necessitates a requirement analysis of the respective use case.

## Virtual data shelf

### Database and use cases

The *virtual data shelf* can facilitate medical research and hypothesis generation by showing and interacting with previously treated cases in a 3D environment. One application area is the analysis of intracranial aneurysms. These are pathological dilatations of blood vessels in the brain. In research, clinical, morphological and hemodynamic parameters are used to predict their rupture risk [13, 14]. Based on this example, the database used for the shelf is a simple *csv* file. There are only two requirements that have to be met. First, each case (represented by a row) has to have a unique ID. Second, one row has to define the type (numeric or categorical) of each parameter (columns).

For each case, a 3D surface model of the intracranial aneurysm and its relevant surrounding vascular structures was available. Besides metadata, hemodynamic and morphological parameters were extracted semi-automatically for each case and saved in the above-described database [15, 16]. Accordingly, a row of the *csv*-database corresponds to one item in the shelf.

### Requirements and functionalities

Discussions with neurosurgeons revealed that they organize patient data in simple table structures on a desktop PC, which makes getting an overview or a meaningful comparison of multiple cases with many parameters difficult. Given the specific use case, the following requirements regarding the layout and functionalities were derived from previous discussions with neurosurgeons:

1. The shelf should provide a good overview of the database. As many cases as possible should be displayed at the same time and large layouts were used for the study. Thereby, occlusion should be avoided.
2. The shelf should provide interactions to explore the whole database as well as single cases.
3. The underlying database and thus the shelf should be scalable.

To meet these requirements, we used a metaphorical design to create an intuitive user experience [17]. Accordingly, the characteristics size, purpose, functionalities, and visual area of the two candidate metaphors *shelf* and *displays* were compared and discussed and finally combined into a *virtual data shelf*.

The first characteristic is *size*. Interactive displays tend to be smaller than shelves, and even when using a very large display like in cinemas, we are used to having the whole screen in view. Shelves instead can be very large, like in warehouses. To meet *R1*, the size of the proposed visualization is based on the shelf metaphor. Consequently, the size is too large to have the whole shelf in the field of view. Thus, it is more difficult to get a quick overview of the currently displayed cases. But displaying more cases simultaneously shows a larger subset, leading to a better overview of the whole database.

**Fig. 1:**
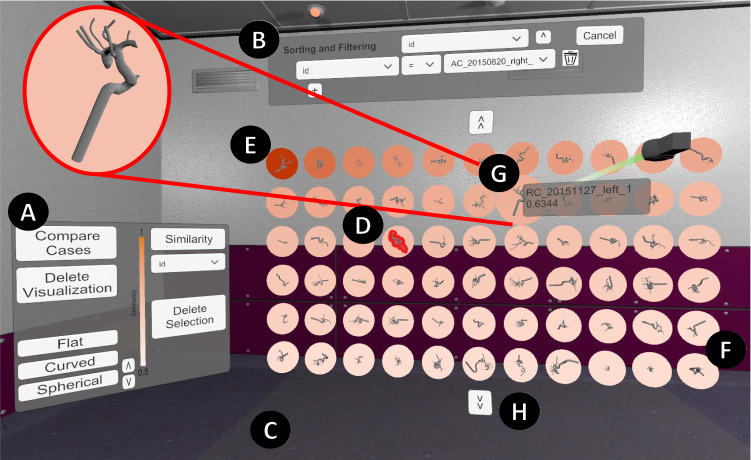
The *virtual data shelf* (*flat*) after similarity calculation. **A** Main Menu, **B** filtering and sorting menu, **C** grab area, **D** selected case, **E** case used for similarity, **F** similarity visualization, **G** info panel, **H** next page. A detailed view of a 3D aneurysm model of the case by **G** with relevant surrounding vessels is in the left-hand corner

When looking at the *purpose*, a display is typically used for visualizing and interacting by clicking on it, whereas a shelf is used for structuring (searching and sorting) cases. However, once a shelf is full, it is usually not changed or restructured frequently.

For the shelf, *functionalities and interactions* are limited to taking out and adding cases. A display and thus programs to work with digital data tables offer various functions such as sorting and filtering. To meet *R2*, both interactions are combined, resulting in the following interaction possibilities: filtering, sorting, and finding similar cases and highlighting, grabbing and taking out. For the first three functionalities, the user has to interact with the user interface (see Fig. 1A, B). Highlighting (see Fig. 1F) an item can be performed by using the trigger button of the VR controller. To grab and take out an item, the user has to point on the item and press the grab button. Is the grabbed item released within the grab area (see Fig. 1C), it automatically returns to its position in the shelf. To leave an item outside the shelf, it has to be released outside of the grab area.

When clicking on a user interface button, the similarity between the currently selected item and all other cases is calculated according to Spitz et al. [18] and visualized via colored circles (similarity is encoded in saturation). This similarity is calculated as Euclidean distance over morphological and hemodynamic parameters as well as the metadata of the cases saved in the database. The user can choose to predict results of any parameter based on the most similar cases to the selected item. Similarity values range from 0 to 1, with 0 meaning no similarity, and 1 being the highest possible similarity when calculating the similarity of a case to itself. To inspect all parameters of cases in detail, a button can be pressed to show a list view of all parameters, and the values of the selected and highlighted cases as a list.

Regarding the *visual area*, the shelf can only hold a limited amount of cases. Although the visual space of a display is limited as well, the amount of cases is unlimited due to scrolling or going to the next page. Since our *data shelf* should use a large database, we enable to use several pages which fulfills *R3*.

For immersion, a semi-realistic room environment was chosen for a feeling of familiarity, as well as to avoid discomfort. The *virtual data shelf* and all its functionalities are displayed and summarized in Fig. 1.

**Fig. 2:**
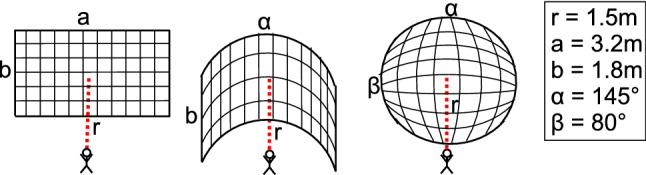
Three layouts: flat, curved and spherical

### Implementation of layouts

The three layouts and their parameters determined by the pilot study are *flat*, *curved* and *spherical* (depicted in Fig. 2). The *flat* layout arose due to the shelf metaphor and the benefit of providing a clear grid alignment enhancing spatial memory [9]. However, sizes are perceived different, and there is perspective distortion at the edges. The second layout is *curved* horizontally, resulting in no perspective distortion and same distances per row if the user is in the center. The third layout is *spherical* and thus curved horizontally and vertically, resulting in all cases having the same distance from the center.

**Table 2:** Characteristics of participants ($n=61$) in *E1*

Age	#	%	Sex	#	%	VR exp	#	%	VG exp	#	%
15-25	19	47.5	M	34	55.74	None	24	39.3	Never	16	26.2
26-35	21	34.4	F	27	44.26	< 15	30	49.2	< 1	12	19.7
36-45	2	3.3				> 15	7	11.5	1-3	7	11.5
46-55	2	3.3							More	26	42.6
56-65	6	9.8									

VR Exp. is experience with VR, VG Exp. is frequency of playing video games per month. # is number of participants with a quality, and % the relative amount of the total number of participants

To ensure the same conditions for all three layouts, they all show the same number of equally scaled items. The gap between the items along straight directions as well as curved directions is consistent across all layouts.

The *virtual data shelf* was implemented with the game engine *Unity*. For the implementation as well as the evaluation, a *HTC Vive Pro Eye* (HTC Corporation, Taiwan) was used.

## Evaluation

For the evaluation, two distances between the user and shelf and a shelf size of $6\,\times \,11$ cases were chosen based on a pilot study with nine participants using the *think-aloud* method. We used two fixed distances $d_1 = r = 1.5\,$m and $d_2 = 2r = 3\,$m as the distance determines how much of the shelf lies within the field of view and how small the objects are. Because of the two tasks, the distance might either support the overview (large distances) or exploration (small distances). Therefore, a small and a large distance was used. However, we made sure that the relevant texture and shape differences of the items are still recognizable when using the large distance.

In the two studies, the users were instructed to sit to keep them from moving (for consistent distances), though results are not expected to change if users stand since the center of the shelf was always placed on the same height as the user’s head. After the preliminary pilot study, evaluation includes two studies, one with non-medical experts and one with experts, to combine qualitative feedback with a statistical analysis.

**Fig. 3:**
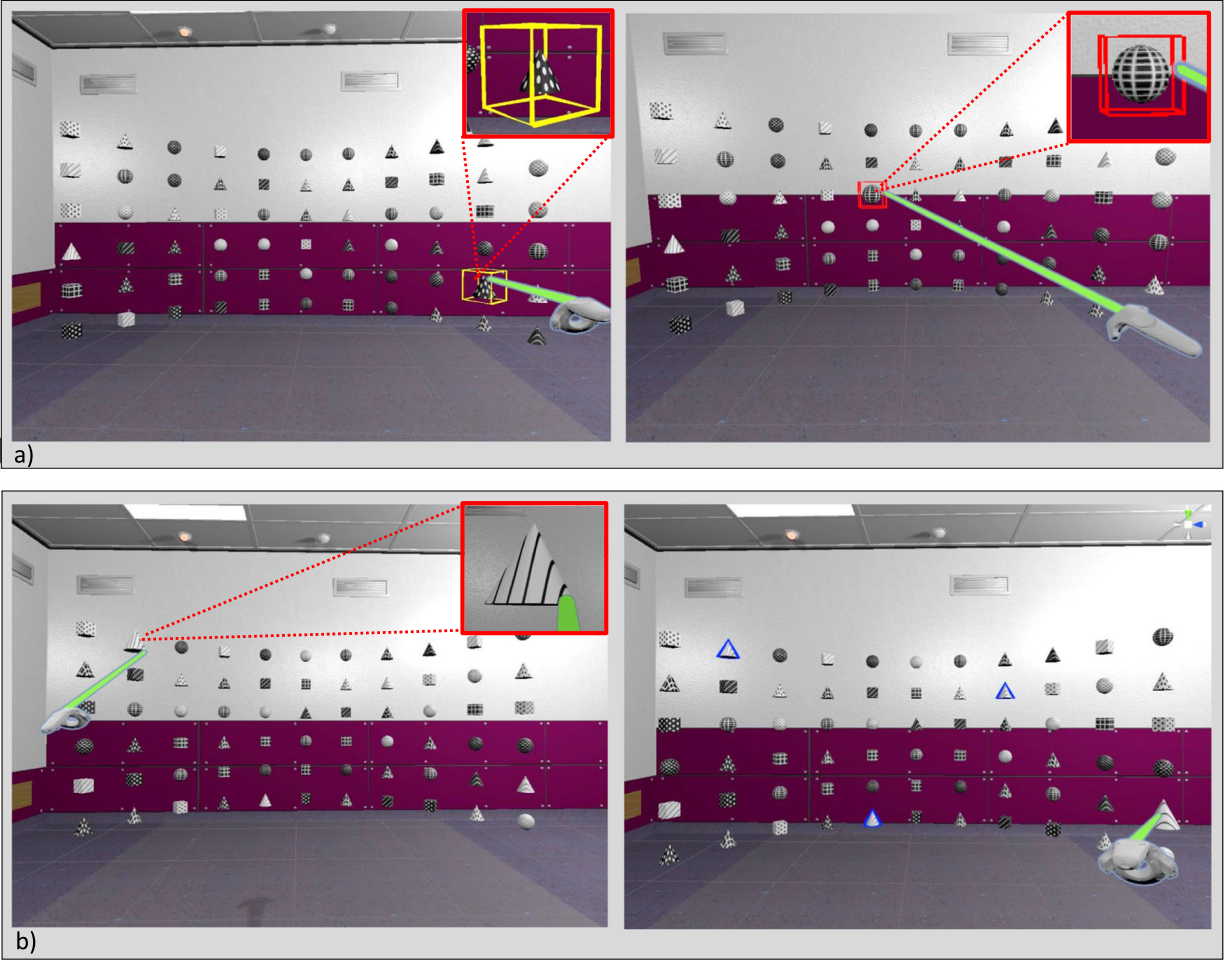
Study procedure: **a** Task 1: pre-attentive search (yellow: first item, red: second item) and **b** Task 2: attentive search. The search items are white cones with black lines. Selected item are highlighted in blue

**Table 3:**
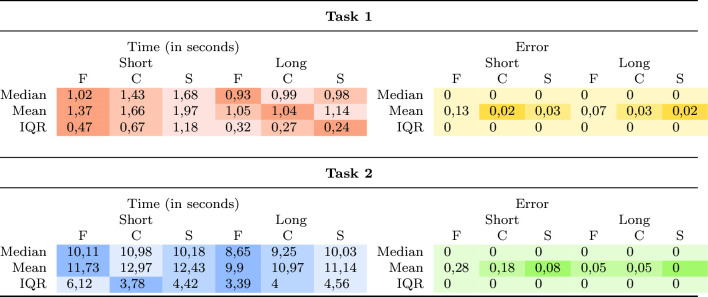
Measured time and error for all three layouts (F = flat, C = curved and S = spherica) and both tasks

The color saturation indicates the order of the results with high saturation equal to best result

### Broad audience study (E1)

In this study, 61 participants (see Table 2) compared the layouts to assess their suitability for exploration and overview tasks using a limited version of the *virtual data shelf*. In this version, only simplified geometries are displayed and all functionalities are removed. Thus, not the target audience, e.g., medical researchers and clinicians, were used as participants, but a broad audience without medical background, in order to assess general overview and exploration capacities. The study consisted of two tasks (see Fig. 3): one for pre-attentive search to assess overview and one for attentive search to assess exploration. After starting the first task, a randomly highlighted object near the border has to be selected. Afterwards, a randomly determined object within a specific Manhattan distance ($d_\textrm{Manhattan} = 7$) is highlighted and has to be selected. Thereby, the time between selecting the first and the second objects as well as errors was captured. This was done twice per layout with the two distances in randomized order. The order of layouts was arranged according to a *balanced Latin square*, a method that reduces order-effects by ensuring an even spread of options across every trial and across the whole study.

In the second task, the participants had to select multiple objects attentively by finding all cones with black lines (see Fig. 3). Here, again the layouts were ordered according to a *balanced Latin square* and for each layout the two user positions were used in a randomized order. For this task, the duration and error were measured. Errors include selecting a wrong item and missing correct items. Since for each subtask a different arrangement was used, two similar arrangements were created and flipped horizontally and vertically, yielding six different but similar arrangements. The order of arrangements also underlies a *balanced Latin square*. Finally, they had to rate the layouts subjectively via a *five-point Likert scale* regarding their suitability to get an overview and to explore. Another questionnaire gathered demographic data. We also asked about experience with video games and VR, as a familiarity with virtual spaces, controllers, and their interactions might be a factor of how a layout is rated. We chose definite cutoff values where we expected confident differences in experience to avoid user uncertainty.

After the tasks were explained to the participant using a rehearsal with one layout, one distance and a pattern which are not used in the study, they had one trial.

### Medical expert evaluation (E2)

Finally, we evaluated the *virtual data shelf* qualitatively with possible users using the *think-aloud* method. We used a database of 76 intracranial aneurysms with up to 34 metadata and morphological and hemodynamic parameters per aneurysm. The main focus of *E2* was on investigating concrete use cases where such a tool could be supportive, including requirements and adaptions that would be necessary to implement. The usability in general, the visualization of similar aneurysms and the possibility to examine and compare cases were also assessed. The layouts were additionally evaluated for the aneurysm-specific use case. Two neuroradiologists and two neurosurgeons participated. The two male neuroradiologists were in the age ranges [36–40] and [46–50], experienced VR less than 15 times, one plays video games several times a week and the other one never plays video games. The neurosurgeons, [46–50] and [51–55] years, have experienced VR less than 15 times and never, and one surgeon plays video games one to three times a month, whereas the other one plays video games less than one time a month.

## Results

### Quantitative results

The quantitative data from *E1* such as *errors* and *time* were statistically analyzed using the *Kruskal–Wallis test*. This test was chosen as the data do not underlie a normal distribution. To further analyze statistical differences, a *pairwise Wilcoxon test* with *Bonferroni correction* was used as post hoc analysis. In total, one data point of the first task, two data points from the second task and one data point of the VR questionnaire were removed due to technical problems and accidentally skipping a subtask. The following results are structured according to the different tasks.

The first task shows significant differences between the layouts when using the short distance ($p<0.001$). The post hoc analysis revealed statistically significant differences between the curved and flat layouts as well as the spherical and flat layout regarding time. The effect size is $f=0.46$ and thus indicates a strong effect. The descriptive results show that regarding time the flat layout has the smallest median, mean, and interquartile range. This is followed by the curved layout when using the short distance. However, regarding the long distance the differences are very small. The exact values are summarized in Table 3. That the flat layout is faster coincides with the results of the study by Takashina et al.’s [12].

**Fig. 4:**
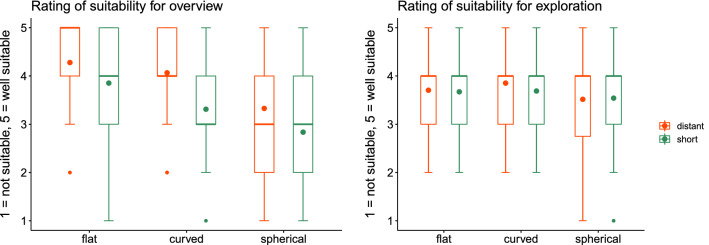
Rating of the layouts from E1 regarding their suitability to get an overview and for exploration

Regarding the second task, there are no significant differences between the layouts regarding time and error (for both distances).

Concerning the questionnaire, the statistical analysis revealed significant differences regarding the suitability to get an overview for short and long distance (both $p<0.001$). The post hoc analysis shows differences for short distances ($f=0.35$) between flat and spherical, and for long distances ($f=0.39$) between flat and spherical as well as curved and spherical. The descriptive results (see Fig. 4) show that, regarding overview, the flat layout is always perceived as the best one and spherical as worst. For flat and curved, the short distance is rated as not as good as the long distance. Regarding exploration, no significant differences could be found.

### Qualitative results

The feedback from *E2* is categorized into: applications and layouts.

*Applications* For the neuroradiologists, such an application would be very useful if it is extended by data about the patient-specific treatment. The treatment of similar cases can guide the treatment decision for a new case, resulting in a more objective decision. Usually, this decision is based on experience and subjective intuition, which can be incomplete, incorrect or biased. Especially since the database contains medical data from various institutions, comparing treatment decisions and results is beneficial. If treatment data is included, the neuroradiologists stated that the shelf can be explored using the described functionalities while also exploring the 3D anatomy of interesting cases. Thereby, they can generate and possibly verify hypotheses such as:

- All aneurysms that were similar (morphologically and/or hemodynamically) before the treatment were treated similarly.
- All similarly treated aneurysms have a similar outcome.
- Different treatment methods of similar aneurysms lead to different outcomes.

One can also expand the *virtual data shelf* and include not only the treatment data of previous cases, but also the possibility to virtually treat the aneurysm and examine the results, for example, based on the blood flow or include MRI data to highlight inflammations.

The neurosurgeons mentioned that the tool in its current state is not applicable to gain new insights into surgery preparation, but a collaborative VR application for patient education might be useful. For this, other anatomical structures such as the skull have to be included to show access planning and describe the surgery. However, one has to examine the acceptance among patients. Besides patient education, the *virtual data shelf* is useful to explore an aneurysm prior to a surgery and to get a better understanding of the anatomical structures. For this, more models including more vessels, the optical nerve, and bones, would be helpful. Furthermore, they mentioned that exploring an aneurysm in VR is much better than exploring it via 3D images. They also need more surrounding structures and would like to explore head data with aneurysms starting from the outside and removing single parts step by step. When the aneurysm is reached, it would be helpful to scale it.

*Layouts* After trying out all three layouts, both neuroradiologists stated that they preferred the spherical layout, although a larger distance (larger than the radius) is necessary to get an overview. With the spherical layout, the models seem to be more tangible due to the equal distances, thus enclosing the user. Because of this, the participants stated that it is more intuitive. Some surgeons emphasized that for a detailed exploration the curved and spherical layout is much more intuitive and appropriate, since all objects have the same distance. One of them also recognized that the spherical layout has the benefit that objects above each other can be differentiated more easily as they differ in their depth. Consequently, it is easier to concentrate on one single item. One slightly preferred the flat layout. Overall, there was no favorite layout.

## Discussion

*E1* revealed that the flat layout is best and fastest to get an overview, especially for small distances. With short distances, all three layouts are too large to be completely in the field of view. However, with the flat layout, the head does not have to be rotated as much (recall Fig. 2). This coincides with the questionnaire results which rated flat best regarding overview. This could be explained by the fact that we are used to work with displays in front of us, being entirely in our field of view. We did not include a questionnaire regarding comfort, which is recommended in future studies to reveal correlations between comfort and layout. VR provides the possibility to use more space and thus displaying and interacting with models that are distributed around the user. Movement is therefore required to fully exploit the potential of VR. Since a lot of our participants had never used VR ($39\%$) or just a couple of times ($49\%$, see Table 2), the lack of familiarity with the medium might have influenced the result. To avoid this, a long-term evaluation is recommended and might provide additional insights. Concerning both the VR experience as well as the frequency of playing video games, no differences regarding the ranking in the questionnaire could be found. One limitation here is the amount of participants in each of the subgroups which does not allow proper statistical analysis.

When looking at the error rate, the flat layout has more errors than the other two layouts. This might be due to the flat interaction angle when selecting objects at the edges.

In contrast to *E1*, where only slight differences occurred with regard to exploration, *E2*, the qualitative feedback, shows that medical professionals prefer the curved or spherical layout for a detailed exploration. This also confirms the results of Liu et al. [8]. We hypothesize that medical experts appreciated being closer to the 3D models, as they were interested in and familiar with the anatomy and thus wanted to inspect individual cases more closely. This is facilitated by the curved and spherical layouts since they enable a closer distance between the user and all models as compared to flat layout. Experience levels with VR and video games across the experts were varied, and we had too few participants for a statistical evaluation, though we found no correlation between performance and experience in general participants in *E1.* Thus, based on our comparison using non-experts and experts, all layouts should be provided and the user should be allowed to change them based on current task and preference.

In general, the *virtual data shelf* is a good basis for medical research. However, for hypothesis generation, our tool has to be further refined and extended. In case of treatment decision, treatment properties have to be identified and quantified to compare and find similar cases. Once these have been included and the database has been enlarged considerably, the neuroradiologists would consider actively using the tool for the mentioned tasks. It can also be extended by including virtual treatment possibilities or combining it with virtual training and treatment applications. Other applications mentioned by the experts are medical education and training of anatomical understanding. Hence, not just the affected structure but additional surrounding structures to provide the necessary anatomical context are required.

In contrast to previous works, we proposed a design combining benefits from the shelf and display metaphor. Furthermore, in our comparison of different layouts, we used much more and complex objects in accordance with our goal of displaying and working with a large database. In contrast to other studies comparing different layouts [12], we could not find statistically significant evidence showing that interacting on a curved layout is faster.

### Limitations and future work

*E1* was conducted with non-experts since the focus was on the layouts and the general exploration and overview tasks. Although a specific use case was selected based on previous feedback from medical experts, the evaluation with them emphasized that the *shelf* has to be expanded and specialized according to the discipline and objective. Accordingly, in the future we want to adapt the shelf to one of the identified medical use cases. Then, a more precise evaluation in which the ability of the shelf regarding data exploration, hypotheses generation and decision making can be evaluated. Therefore, specialized evaluation tasks have to be created. To investigate the benefit of such a *virtual data shelf*, an evaluation over time with more respective experts is highly recommended.

Another possible extension would be a focus on arrangement and grouping of models in relation to each other, making use of distances and depth, thus exploiting the entirety of the VR space.

Since our focus was the comparison of the layouts, we used a fixed number of items based on *R1* and the pilot study. However, a larger evaluation regarding information overload and the preferred number of items could give more insights.

There are studies highlighting advantages of VR over desktop when dealing with complex anatomical 3D structures [1–3]. We did not include a comparison of the two modalities as our focus was on the comparison of different layouts within VR; however, a future study to investigate the new and the conventional modality might be interesting. Other future studies could address ergonomics, where the virtual shelf’s position, height, width and orientation are specifically scaled to each user’s individual needs and comfort based on their size and preference.

## Conclusion

Our studies show that an appropriate arrangement of 3D objects in an immersive virtual reality environment depends on the user and task objectives. If getting an overview as well as an exploration is required in an application, the users should have the possibility to change the layouts according to their needs.

The proposed *virtual data shelf*, which combines benefits of virtual shelves and displays, serves as a good basis for various applications such as in the medical field and can easily be extended by additional functionalities.
